# Moderate and Vigorous Physical Activity Intensity Cut‐Points for Hip‐, Wrist‐, Thigh‐, and Lower Back Worn Accelerometer in Very Old Adults

**DOI:** 10.1111/sms.70009

**Published:** 2025-01-03

**Authors:** Mathias Skjødt, Jan Christian Brønd, Mark A. Tully, Li‐Tang Tsai, Annemarie Koster, Marjolein Visser, Paolo Caserotti

**Affiliations:** ^1^ Department of Sports Science and Clinical Biomechanics University of Southern Denmark Odense Denmark; ^2^ Department of Sports Science and Clinical Biomechanics, Center for Active and Healthy Ageing (CAHA) University of Southern Denmark Odense Denmark; ^3^ School of Medicine Ulster University Londonderry UK; ^4^ Research Unit for ORL—Head and Neck Surgery and Audiology Odense University Hospital and University of Southern Denmark Odense Denmark; ^5^ Department of Social Medicine, CAPHRI Care and Public Health Research Institute Maastricht University Maastricht the Netherlands; ^6^ Department of Health Sciences, Faculty of Science, Amsterdam Public Health Research Institute Vrije Universiteit Amsterdam Amsterdam the Netherlands

**Keywords:** classification, machine learning, validation, VO_2_Reserve, wearable devices

## Abstract

Physical activity (PA) reduces the risk of negative mental and physical health outcomes in older adults. Traditionally, PA intensity is classified using METs, with 1 MET equal to 3.5 mL O_2_·min^−1^·kg^−1^. However, this may underestimate moderate and vigorous intensity due to age‐related changes in resting metabolic rate (RMR) and VO_2_max. VO_2_reserve accounts for these changes. While receiver operating characteristics (ROC) analysis is commonly used to develop PA, intensity cut‐points, machine learning (ML) offers a potential alternative. This study aimed to develop ROC cut‐points and ML models to classify PA intensity in older adults. Sixty‐seven older adults performed activities of daily living (ADL) and two six‐minute walking tests (6‐MWT) while wearing six accelerometers on their hips, wrists, thigh, and lower back. Oxygen uptake was measured. ROC and ML models were developed for ENMO and Actigraph counts (AGVMC) using VO_2_reserve as the criterion in two‐third of the sample and validated in the remaining third. ROC‐developed cut‐points showed good‐excellent AUC (0.84–0.93) for the hips, lower back, and thigh, but wrist cut‐points failed to distinguish between moderate and vigorous intensity. The accuracy of ML models was high and consistent across all six anatomical sites (0.83–0.89). Validation of the ML models showed better results compared to ROC cut‐points, with the thigh showing the highest accuracy. This study provides ML models that optimize the classification of PA intensity in very old adults for six anatomical placements hips (left/right), wrist (dominant/non‐dominant), thigh and lower back increasing comparability between studies using different wear‐position.

**Clinical Trial Registration:** clinicaltrials.gov identifier: NCT04821713

## 1 Introduction

Physical activity (PA) is associated with a reduced risk of negative mental and physical health outcomes in older adults [1, 2] and it is therefore widely recognized as one of the most important lifestyle factors to incorporate in preventive strategies. PA guidelines recommend 150–300 min of moderate intensity or 75–150 min of vigorous intensity per week [2]. Regardless, recent studies have indicated that even small (4–20 min) weekly periods of vigorous PA, assessed with accelerometers, can significantly reduce the risk of morbidity and mortality [3]. These new findings raise the need of precisely classifying both moderate and vigorous intensity. The classification of PA intensity in older adults is challenged by age‐related physiological changes which include the decline in resting metabolic rate (RMR) and maximum aerobic capacity (V̇O_2_max).

A recent systematic review has shown RMR to be approximately 25% lower in older adults compared to the standard of one MET (3.5 mL O_2_·min^−1^·kg^−1^) assumed in adults [4]. Therefore, age‐related changes in RMR, should be considered when classifying PA intensities and the 1 MET value should be adapted to the older population. Few studies have considered age‐related changes in RMR when developing PA cut‐points for accelerometers using the METs metric [5, 6, 7, 8, 9]. Despite the relevance of this approach, it raises a few concerns. The new compendium of PA for older adults [10] calls for more research to determine if adjusting for age‐related changes in RMR (e.g., 2.7 mL O_2_·min^−1^·kg^−1^), while using 3 and 6 METs cut‐points for moderate and vigorous intensity, is appropriate. Indeed, the RMR‐adjusted MET approach does not consider the substantial decline of the V̇O_2_max, which has been shown to decline up to 25% per decade after the age of 60 years [11]. Such decline causes an increase in the relative intensity for a given activity [12, 13], so V̇O_2_max would also be important to consider when classifying PA intensity. One way to consider the age‐related changes in both RMR and V̇O_2_max is by V̇O_2_Reserve (V̇O_2_R) defined as the difference between V̇O_2_max and RMR [14]. V̇O_2_R may provide an equal comparison of relative intensities in older adults with different V̇O_2_max and provide a more accurate intensity classification.

Accelerometers have been increasingly used to objectively assess PA level in the past couple of decades [15]. The raw accelerometer output is often translated from its biomechanical properties into physiological measure such as energy expenditure. A common way to analyze device‐based outcome is by using accelerometer cut‐points derived from Metabolic Equivalent of Task (MET) to classify light (1.5–2.9 METs), moderate (3–5.9 METs) and vigorous (> 6 METs) PA intensities for older adults [5, 6, 7, 8, 9, 16, 17, 18]. Regardless, the current METs intensity classification may, however, underestimate moderate‐to‐vigorous physical activity (MVPA) because it does not account for the age‐related decline in RMR and V̇O_2_max, particularly in the very old population [19, 20].

Accordingly, two recent studies have suggested the use of relative rather than absolute cut‐points to account for the physiological decline with age [21, 22]. The findings from these studies provide a novel paradigm that the percentage of time spent in MVPA may not decrease at older age as consistently reported. For example, one study showed an increase in MVPA with age when using heart rate reserve to classify PA intensity and compared to absolute cut‐points from chest‐worn accelerometry in older adults [22]. The findings highlight the need of tailoring intensity PA cut‐points for older adults which incorporate the physiological changes in RMR and V̇O_2_max.

Receiver Operating Characteristics (ROC) curve analysis and linear regression are commonly used to develop accelerometer intensity cut‐points in older adults [5, 6, 7, 8, 9, 16, 17, 18]. One of the main advantages of ROC analysis is that it does not assume a linear relationship between acceleration and energy expenditure, but it cannot adjust for co‐variates such age, sex, or physical function. Machine learning (ML) offers a potential alternative, as it can handle non‐linear elements and adjust for various relevant variables [23] which might increase the accuracy of the PA intensity classification.

Besides the criterion measure and various statistical models, the anatomical placement of the accelerometer also impacts the established cut‐points. For instance, accelerometers worn on the wrist and hip during the same activity with identical energy demand provide different acceleration outputs [8, 9, 16]. Therefore, to enhance comparability between studies using accelerometers at different anatomical placements in older adults, it is essential to establish cut‐points using multiple wear locations during the same activity protocol.

Overall, the objectives of this study were to establish moderate and vigorous accelerometer cut‐points for hip (left and right), wrist (dominant and non‐dominant), thigh, and lower back using ROC analysis, and compare them to ML classification models using V̇O_2_R as the criterion in community‐dwelling older adults, aged 75 years and older.

## 2 Method

### 2.1 Design and Participants

The data used in this study have been collected as part of the Energetics in Old Age (ENGAGE) Study which is a cross‐sectional study including both free‐living and laboratory measurements. ENGAGE is part of a larger Horizon2020 project named PROMISS (Prevention Of Malnutrition In Senior Subjects) and was carried out between August 2019 and June 2022 and has received ethical approval from The Regional Scientific Ethical Committees for Southern Denmark (S‐20170150) and was registered in clinicaltrials.gov (NCT04821713). A total of 98 community‐dwelling Danish older adults were recruited by various channels (e.g., nationally regulated preventive home visits or announcement in newspapers). Inclusion criteria were (1) older than 75 years, (2) intact cognitive function measured by the SF‐MMSE [24] (score > 3) and (3) passed medical check with the main purpose to exclude participants with uncontrolled cardiovascular, pulmonary or metabolic disease. Participants were also excluded if they were medically unstable with the potential to affect the metabolism (e.g., cancer, severe organ disease, bed‐ridden). All participants received written information about the project and the measurements and signed a written consent form.

Eligible participants were invited to attend 3 days of laboratory assessments within a 14‐days period. The first day (day 1) contained measurements of anthropometrics and RMR. The second day (between day 4 and day 12) consisted of synchronized oxygen uptake and accelerometry measurements during an activity protocol including two circuit of Activities of Daily Living (ADL) and two 6‐min‐walking‐test (6‐MWT) with self‐selected and maximal gait speed. On the third assessment day (day 14) the participants performed a V̇O_2_max test. Among the 98 participants, 76 were included in the current study, as they had complete data for the criterion measure (i.e., V̇O_2_R).

### 2.2 Measurements

#### 2.2.1 ADL Activities

The protocol for daily activities consisted of two semi‐standardized circuits which were estimated to have different metabolic intensities. Both circuits were designed to mimic common activities of daily life carried out in a “common apartment size”. The activities were thereby placed within an area of 5–10‐m walking distance between each activity and participants were asked to perform the activities at their own self‐selected pace. A 10‐min steady state period for each activity was provided before the collection of expired gas was started. The first circuit (ADL Circuit 1) consisted of five daily activities: (1) dressing and undressing; (2) personal care (simulating combing hair and brushing teeth); (3) setting and clearing a table (4) light upper body sweeping and (5) simulating food preparation. The second circuit (ADL Circuit 2) consisted of four daily activities: (1) making the bed; (2) doing laundry; (3) vacuuming cleaning; and (4) washing windows.

#### 2.2.2 Self‐Selected and Maximal 6‐Minutes‐Walking‐Test (6‐MWT)

In the 6‐MWT participants were asked to walk around a squared 33‐m track first at a self‐selected, and after a minimum break of 5 min, at maximal gait speed. Before each test heart rate was assessed to ensure individual values similar to quite standing position (±5 beats/min). Expired gas was collected during the last 3 min of the each 6‐MWT.

### 2.3 Oxygen Uptake (V̇O_2_)

V̇O_2_ was assessed by the gold standard Douglas bag method [25] for all activities except the incremental V̇O_2_max test for which a computerized system (O2CPX, Oxigraf, USA, version 8.02, Innocor, Denmark) was used (*see below*) and it has been earlier reported [19].

Briefly, during ADL and walking activities the Douglas bag was connected to a portable system moved around by a research assistant. For measurement of V̇O_2_, subjects worn a facemask (V2 mask, Hans Rudolph, IL, USA) connected to a two‐way non‐rebreathing valve (Innovision A/S) and two tubes one for inhaling and one for exhaling. After a steady‐state period the expired air was collected, and the content analyzed within a short while to avoid gas diffusion. The gas analyzer (INN00400, Innocor, Denmark) was calibrated with a two‐point calibration before the measurement of O_2_ and CO_2_ in each Douglas bag. The bag was emptied trough a gas spirometer pump (Rayfield Equipment, Vermont, USA) and the volume of expired air were calculated. The gas spirometer was previously validated by placing it in series with a 120 L Tissot spirometer.

### 2.4 Accelerometry

#### 2.4.1 Laboratory Assessment

During the laboratory measurements of ADL and 6MWT (the second assessment day), research personnel attached six accelerometers on participants' left and right hip (on iliac crest with a strap; device model: Actigraph GT3X+), dominant and non‐dominant wrists (device model: GENEActiv), right thigh (mid‐point between femur greater trochanter and patellar bone in a sitting position; device model: Axivity AX3) and lower back (on the skin area above waistline to the right of the spine; device model: Axivity AX3). Different accelerometers were selected to represent some of the most common brands for each specific placement when the study was designed. A recent study has shown good to excellent intraclass correlations between the intensity gradients of the three included accelerometers [26]. The sampling frequency was set at 100 Hz and all accelerometers were synchronized. At the end of the activity protocol accelerometer data were downloaded and saved as raw data. The Actigraph files were downloaded with the ActiLife software version 6.13.4, the GENEActiv files with the GENEActiv software version 3.3 and the Axivity files with the OmGui software version 1.0.0.43. All raw accelerometry data were calibrated using the method proposed by Van Hees et al. [27]. From the raw calibrated acceleration multiple time series features were calculated using a non‐overlapping window length of 60 s. The features generated include the Euclidean norm minus one (ENMO) [27] and actigraph vector magnitude counts (AGVMC) [28], mean acceleration of the individual axis (maccX, maccY, maccZ) and standard deviation (sdaccX, sdaccY, sdaccZ) in each axis (x, y, z), mean, standard deviation and maximal standard deviation of the vector, mean and standard deviation of inclination (incl/sdincl) and angel (angl/sdangl) were calculated (Table S1). The Mean Amplitude Deviation [29] and Activity Index [30] were also computed, with the findings detailed in the Tables S2 and S3. Protocol start and stop time was used to identify the activity specific periods during the recording and thus to extract and synchronize accelerometer data with V̇O_2_ measurements. All raw accelerometry processing was done in Matlab (Version 9.9.0 R2023b, Mathworks Inc., Natick, MA, USA).

#### 2.4.2 Resting Metabolic Rate

RMR was assessed in the morning after an overnight fast of 12 h including refraining from exercise and smoking. Participants were resting in a supine position in a quiet room with deemed light and comfortable temperature. The collection of expired gas started 30 min after quiet laying to ensure steady state.

#### 2.4.3 V̇O_2_max

V̇O_2_max was assessed with an incremental treadmill walking test measured by a computerized mixing chamber system (O2CPX, Oxigraf, USA, version 8.02, Innocor, Denmark). The gas and flow analyzers were calibrated before the assessment with a two‐point calibration of known gas concentrations and a 3‐l syringe, respectively. For familiarization to the treadmill the participants performed a short warmup before the V̇O_2_max test. Participants walked with a constant comfortable self‐selected walking speed throughout the entire test. The protocol for selecting the walking speed is described elsewhere [19]. During the V̇O_2_max test the slope was increased every second minute starting at 0% inclination followed by 5%, 10%, 12% inclinations and continuing with a 2% increase every second minute until exhaustion. Heart rate was continues monitored during the test and Rate of Perceived Exertion (RPE) was reported immediately after completion of the test. V̇O_2_ data were provided for each 10‐s intervals. V̇O_2_max was calculated as the three highest consecutive 10‐s intervals. Participants were given strong verbal encouragement during the test to achieve V̇O_2_max, which was defined valid if two out of three criteria were reached: (1) heart rate (HR) within 10 beats/min of age‐predicted maximal HR (220‐age), (2) respiratory exchange ratio > 1.10, (3) a Borg scale score (RPE) of ≥ 17.

### 2.5 Anthropometrics

On the first day anthropometric and body composition measurements were obtained. Height was accurately measured using a standard stadiometer (SECA, Germany) up to the nearest 0.1 cm. The bioelectrical impedance analysis (BIA) device (TANITA, model BC‐420MA) was used to measure weight and body composition. An estimated weight of the clothes (0.7 kg) was subtracted from the total weight measurement.

### 2.6 The Short Physical Performance Battery (SPPB)

Participants physical function of the lower extremities was evaluated by the Short Physical Performance Battery (SPPB) which incorporates balance, gait, and the ability to rise from a chair. Each item is scored with a scale which ranges from 0 to 4 points. Each score is finally summed to obtain a total score (range from 0 to 12 points) [31].

### 2.7 Data Analysis

#### 2.7.1 Classification of PA Intensity by % of V̇O_2_R

The V̇O_2_ values from each activity were converted into % of V̇O_2_R defined as the difference between RMR and V̇O_2_max [14]. The percentages of V̇O_2_R were calculated by the following formula:

$$\%VO_{2}R=\frac{{\mathrm{VO}}_{2}\text{Activity}-{\mathrm{VO}}_{2}\mathrm{RMR}}{{\mathrm{VO}}_{2}\max-{\mathrm{VO}}_{2}\mathrm{RMR}}\times 100$$

Intensity cut‐points based on the % of V̇O_2_R were obtained from the recommendations of *American College of Sports Medicine* using light (< 40% V̇O_2_R), moderate (40%–59.9% V̇O_2_R) and vigorous (≥ 60% V̇O_2_R) [32].

## 3 Statistical Analysis

All statistical analyses were carried out using SPSS (version 29.0.1.0, Chicago SPSS Inc.) and R statistical software (version 4.4.0., Vienna, Austria). Continuous variables of the participants characteristics (Table 1) are presented as mean and standard deviation and categorical variables are presented as frequencies. Furthermore, boxplots were developed to illustrate the absolute and relative changes in ENMO, AGVMC, VO_2_ and %VO_2_R across the four protocol activities. Boxplot with edges of the box representing the first and third quartile. The whiskers extend from the edges of the box to the smallest and largest values within 1.5 times the inter quartile range from quartile 1 and 3, respectively.

**TABLE 1:** Participants characteristics.

	All (*n* = 77)	Training sample (*n* = 50)	Validation sample (*n* = 26)
Variable	Mean ± SD	Mean ± SD	Mean ± SD
Age (years)	80.2 ± 3.7	80.6 ± 2.4	79.6 ± 4.3
Sex (female, %)	43	47	35
Number of chronic diseases	2.5 ± 1.5	2.4 ± 1.5	2.8 ± 1.6
Height (cm)	169 ± 9	169 ± 10	171 ± 8
Body weight (kg)	73.3 ± 13	72.9 ± 14	74.1 ± 12
BMI (kg·m^‐2^)	25.4 ± 3.2	25.4 ± 3.2	25RMR.4 ± 3.2
FM (%)	28.8 ± 6.9	29.3 ± 6.9	27.8 ± 7.0
FFM (%)	71.2 ± 6.9	70.7 ± 6.9	72.1 ± 7.0
SPPB (score)	11.3 ± 1.3	11.4 ± 1.1	11.1 ± 1.6
6‐MWT—Self (m·s^−1^)	1.15 ± 0.18	1.14 ± 0.15	1.17 ± 0.21
6‐MWT—Max (m·s^−1^)	1.52 ± 0.22	1.52 ± 0.21	1.53 ± 0.25
RMR (ml O_2_·min^−1^·kg^−1^)	2.7 ± 0.4	2.6 ± 0.3	2.7 ± 0.4
VO_2_—ADL Circuit 1 (ml O_2_·min^−1^·kg^−1^)	8.9 ± 1.3	8.9 ± 1.3	8.9 ± 1.4
VO_2_—ADL Circuit 2 (ml O_2_·min^−1^·kg^−1^)	10.7 ± 1.6	10.6 ± 1.6	10.7 ± 1.7
VO_2_–6‐MWT—Self (ml O_2_·min^−1^·kg^−1^)	12.9 ± 1.8	12.9 ± 1.7	12.9 ± 1.9
VO_2_–6‐MWT—Max (ml O_2_·min^−1^·kg^−1^)	18.6 ± 3.4	18.4 ± 3.1	19.0 ± 3.9
VO_2−_Max (ml O_2_·min^−1^·kg^−1^)	24.1 ± 5.5	24.0 ± 5.0	24.2 ± 6.4
VO_2−_Reserve (ml O_2_·min^−1^·kg^−1^)	21.5 ± 5.4	21.5 ± 4.9	21.5 ± 6.3

Abbreviations: 6‐MWT—Max = 6‐min‐walking‐test at maximal gait speed, 6‐MWT—Self = 6‐min‐walking‐test at self‐selected gait speed, ADL = activities of daily living, BMI=body mass index, FFM = fat‐free‐mass, FM = fat mass, SD=standard deviation, VO_2_ = oxygen uptake.

### 3.1 Establishing Moderate and Vigorous Intensity Cut‐Points and ML Models

Sixty‐seven participants were randomly divided into a training (*n* = 50) and validation dataset (*n* = 26). The training dataset was employed to develop PA intensity cut‐points and ML models, which were subsequently validated using a hold‐out‐sample.

#### 3.1.1 Receiver Operating Characteristics (ROC) Curve Analysis

ROC curve analysis was used to determine PA cut‐points of moderate and vigorous intensity. The accelerometer PA intensities for ENMO and AGVMC were established using (% V̇O_2_R) as the criterion variable, which was computed into a binary variable. For the moderate cut point values < 40% V̇O_2_R was categorized as 0 and values > 40% V̇O_2_R was categorized as 1. For the vigorous cut point values < 60% V̇O_2_R was categorized as 0 and values ≥ 60% V̇O_2_R was categorized as 1. Area under the curve (AUC) was calculated for each ROC analysis as an indicator of the accuracy and Youden's index, that maximizes the sum of the sensitivity and specificity, was used to identify the relevant cut‐points [33].

#### 3.1.2 Machine Learning

There are numerous ML methods which provides the ability to accurately model non‐linear nature of data. However, it is important to select a ML method which balance both complexity and generalizability. In this study the PA intensity classification was developed using random forest model which provides a reasonable flexibility to model non‐linearity but in addition provides important control to avoid overfitting and ensure generalizability [23]. Random forest models build multiple decision trees and combine them to improve the accuracy and robustness of the predictions. Each tree is constructed using a different bootstrap sample of the data and a random subset of features at each split. Based on the included features in the random forest models (Table S1), each node was split using either 2 or 3 features. The best performing model was then used for further analysis. The final model averages the predictions of all trees to reduce variance and avoid overfitting [23]. The models were trained using the caret package and a five‐fold cross‐validation. The models were evaluated using accuracy, sensitivity, and specificity metrics derived from a 5‐fold cross‐validation process (Table 3). In this process, the training dataset was divided into five folds. The model was trained on four folds and validated on the remaining fold. This procedure was repeated five times. The reported accuracy, sensitivity, and specificity are the averages across all five cross‐validations, reflecting the ability to predict PA intensity classification in new data. A model for each anatomical placements was developed using multiple accelerometer features and a priori decided age, sex and BMI (Table S1). The feature importance for each specific model is presented in the Figure S3. The features are ranked with the most important at the top, and the x‐axis represents the importance in percentages, ranging from 0% to 100%.

#### 3.1.3 Validation

In the current study the cut‐points from the ROC analysis and ML models were validated using a hold‐out‐sample. The prediction of PA intensity by these cut‐points and models was evaluated against %V̇O_2_R classification using a multiclass approach. The moderate and vigorous cut‐points from the ENMO metric, determined through ROC analysis, were used in the validation. ENMO was selected due to better performance compared to the cut‐points from AGVMC (Table S4). Similarly, the ML models were used in the validation. Confusion matrices were produced to calculate the overall accuracy of the model, along with class‐specific (i.e., light, moderate and vigorous) precision, sensitivity, specificity, and F1‐scores. The following formulas were used:

$$\text{Accuracy}=\frac{\text{True positive}}{\mathrm{All}\;\text{instances}}$$

$$P\text{recision}=\frac{\text{True positive}}{\text{True positive}+\text{False positive}}$$

$$S\text{ensitivity}\;\left(\text{recall}\right)=\frac{\text{True positive}}{\text{True positive}+\text{false negative}}$$

$$\text{Specificity}=\frac{\text{True negative}}{\text{True negative}+\text{false positive}\;}$$

$$F1\;\text{score}=2\;x\;\frac{\text{Precision}\;x\;\text{Recall}}{\text{Precision}+\text{Recall}}$$

## 4 Results

### 4.1 Characteristics of the Participants

The participants characteristics for all, training sample and validation sample are shown in Table 1. On average participants were 80.2 ± 3.7 years‐old (43% female), had a BMI of 25.4 ± 3.2 kg·m^‐2^, a body fat percentage and a fat‐free mass of 28.8% ± 6.9% and 71.2% ± 6.9%, respectively, and had 2.5 ± 1.5 chronic conditions. Participants were well‐functioning, with a mean SPPB total score of 11.3 ± 1.3 and a 6‐MWT gait speed of 1.15 ± 0.18 and 1.52 ± 0.22 m/s for self‐selected and maximal speed, respectively (Table 1). No relevant or statistically significant differences were observed between the training and validation samples.

### 4.2 V̇O_2_, %V̇O_2_R, ENMO and AGVMC

**FIGURE 1:**
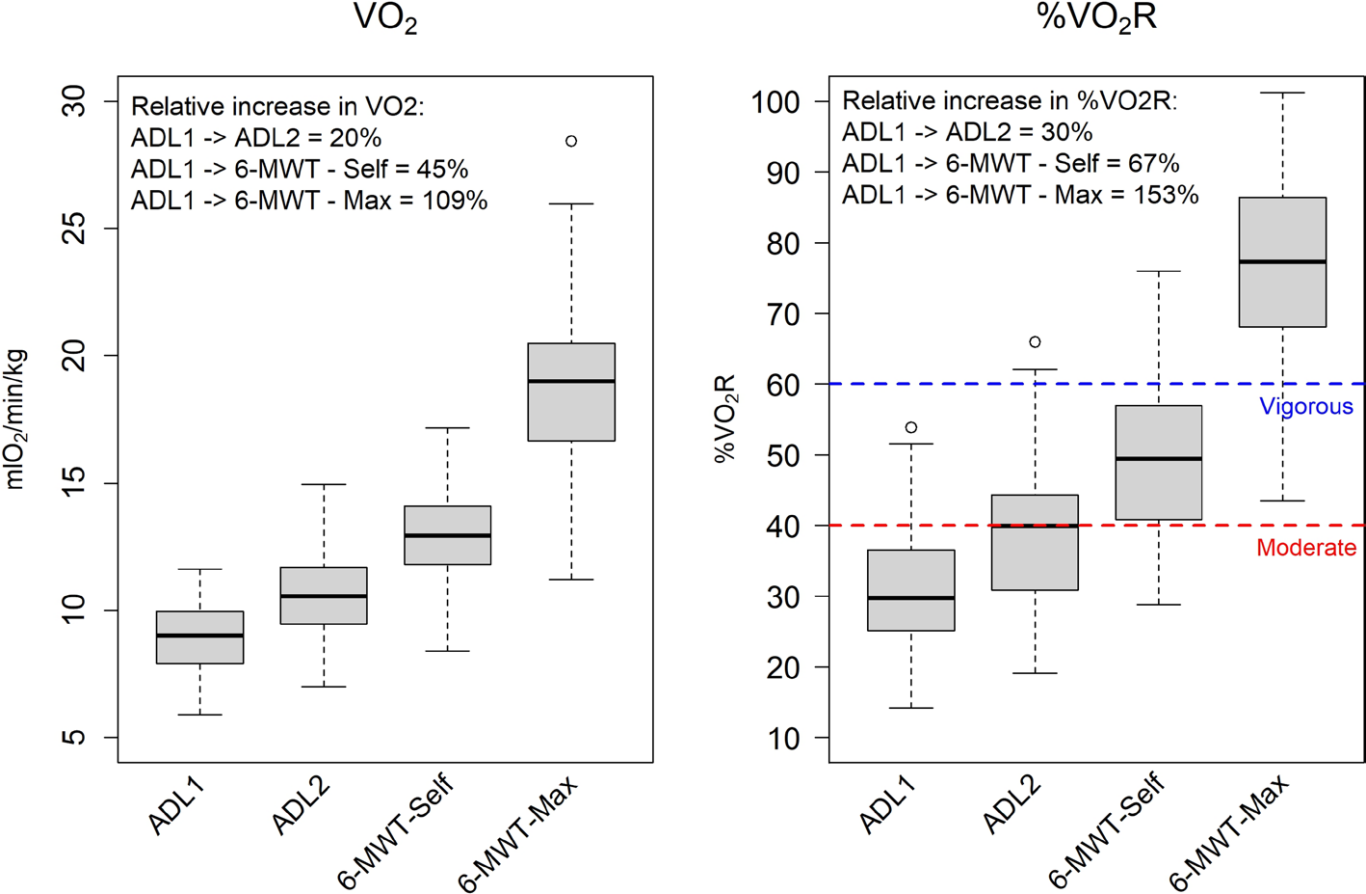
Boxplot of VO_2_ and %VO_2_R by protocol activity. Boxplot with edges of the box representing the first and third quartile. The whiskers extend from the edges of the box to the smallest and largest values within 1.5 times the IQR from Q1 and Q3, respectively; 6‐MWT—Self = 6‐min‐walking‐test at self‐selected gait speed, 6‐MWT—Max = 6‐min‐walking‐test at maximal gait speed, ADL = activities of daily living, VO_2_Reserve intensity cut‐points for moderate and vigorous intensity obtained from American College of Sports Medicine, red = moderate, blue = vigorous.

**FIGURE 2:**
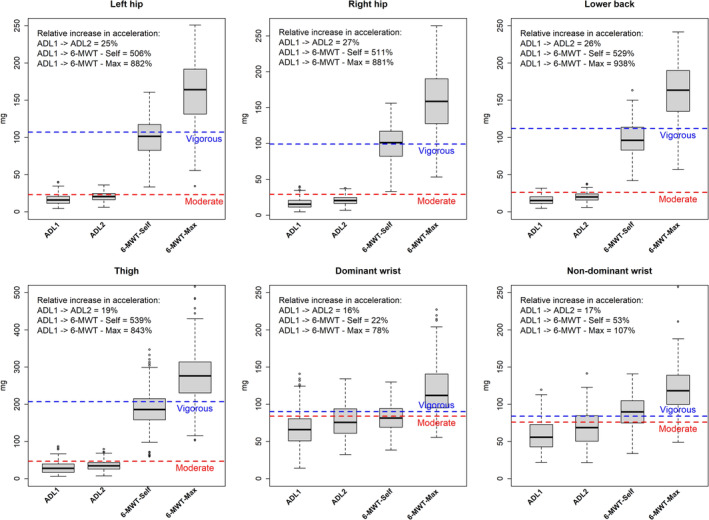
Boxplot of the ENMO acceleration by protocol activity including ROC analysis cut‐points of moderate and vigorous intensity for each anatomical placement (*n* = 76). Boxplot with edges of the box representing the first and third quartile. The whiskers extend from the edges of the box to the smallest and largest values within 1.5 times the IQR from Q1 and Q3, respectively; 6‐MWT—Max = 6‐min‐walking‐test at maximal gait speed, 6‐MWT—Self = 6‐min‐walking‐test at self‐selected gait speed, ADL = activities of daily living, mg = milli gravity, ROC analysis cut‐points for moderate and vigorous intensity, red line = moderate; blue line = vigorous.

**FIGURE 3:**
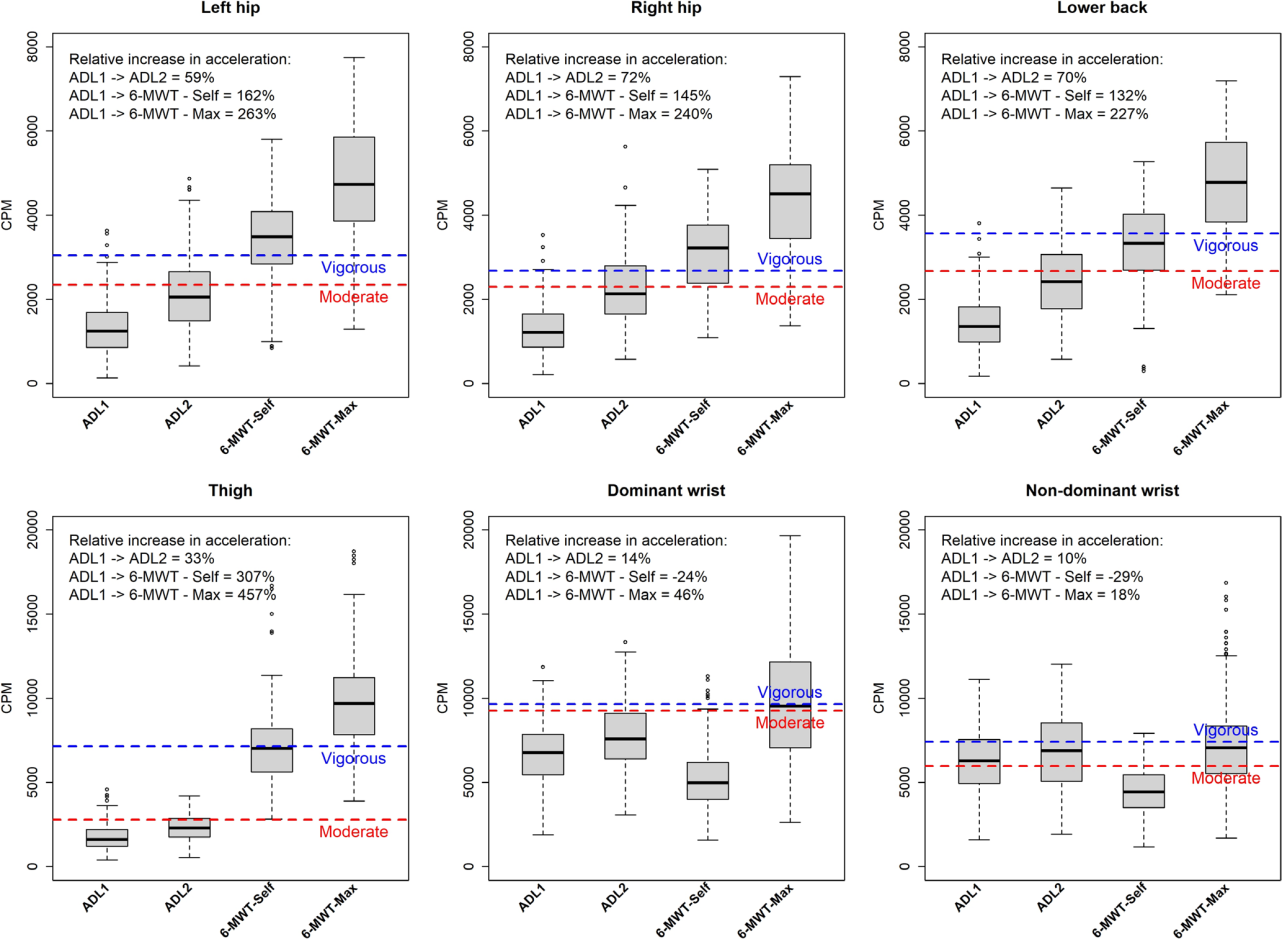
Boxplot of CPM by protocol activity including ROC analysis cut‐points of moderate and vigorous intensity for each anatomical placement (*n* = 77). Boxplot with edges of the box representing the first and third quartile. The whiskers extend from the edges of the box to the smallest and largest values within 1.5 times the IQR from Q1 and Q3, respectively; 6‐MWT—Max = 6‐min‐walking‐test at maximal gait speed, 6‐MWT—Self = 6‐min‐walking‐test at self‐selected gait speed, ADL = activities of daily living, CPM = counts per minute, ROC analysis cut‐points for moderate and vigorous intensity, red line = moderate, blue line = vigorous.

Boxplots of V̇O_2_, %V̇O_2_R, ENMO and AGVMC for hip‐, wrist‐, thigh‐, and lower back‐worn accelerometers during ADL and walking activities are shown in Figures 1, 2, 3. ADL Circuit 1 had the lowest median intensity at 30% (IQR: 25%–37%) of V̇O_2_R, which gradually increased across the four activities. The 6‐MWT at maximal gait speed had the highest intensity at 77% (IQR: 68%–86%) of V̇O_2_R. The relative increases from ADL1 to ADL2, ADL1 to 6‐MWT‐Self, and ADL1 to 6‐MWT‐max were 30%, 67%, and 153%, respectively (Figure 1).

#### 4.2.1 ENMO

The median acceleration from left and right hip, lower back and thigh showed a different pattern compared to %VO_2_R across the four activities. The relative increase between ADL1 and ADL2 was similar to %VO_2_R. However, the relative increase between ADL 1 and the two 6‐MWTs was notably larger compared to %VO_2_R. Specifically, the relative increase in acceleration from ADL 1 to the two 6‐MWTs was approximately 500% and 900% (Figure 2). Additionally, the wrist‐worn accelerometers showed much lower increases between ADL 1 and the walking activities. The median accelerations from the left hip, right hip, and lower back were comparable across all four activities when measured using the ENMO metric. The thigh‐worn accelerometer recorded the highest accelerations during the two 6‐MWTs, while the dominant and non‐dominant wrists showed the highest accelerations during ADL 1 and 2.

#### 4.2.2 AGVMC

The median CPM of both hips and the lower back gradually increased across the four activities. However, there was a higher relative increase between the two ADL circuits and a lower relative increase between ADL 1 and the two 6‐MWTs when compared to ENMO (Figure 3). The dominant and non‐dominant wrists showed a different pattern compared to ENMO. The CPM of ADL Circuit 1 and 2 demonstrated higher values in contrast to the CPM from the 6‐MWT at self‐selected gait speed. Thigh and dominant wrist produced similar median CPM during the 6‐MWT with maximal gait speed.

### 4.3 Moderate and Vigorous Cut‐Points From ROC Analysis

In Table 2, moderate and vigorous cut‐points for PA intensity derived from ROC analysis are presented. The cut‐points established for the left and right hip, lower back, and thigh demonstrated good to excellent AUC values (ranging from 0.84 to 0.93) for both the ENMO and AGVMC metrics. The wrist specific cut‐points yielded lower AUC values, particularly when based on AGVMC (0.54–0.69). The cut‐points for moderate and vigorous PA intensity were similar across the left hip, right hip, and lower back when using the ENMO metric. Specifically, moderate intensity fell within the range of 23–29 mg, while vigorous intensity was between 99 and 112 mg. However, the cut‐points derived from the thigh were notably higher, with moderate intensity at 47 mg and vigorous intensity at 207 mg. The ENMO cut‐points for moderate and vigorous intensity were relatively similar for both the dominant and non‐dominant wrists. For AGVMC, the difference between moderate and vigorous cut‐points was smaller compared to ENMO, with the dominant and non‐dominant wrists showing a similar pattern as for ENMO. The right and left hip showed similar cut‐points for moderate (2299 vs. 2343 CPM), but different cut‐points for vigorous (2681 vs. 3046 CPM). Additionally, the moderate cut point for the thigh‐worn accelerometer was similar to those for the lower back (2794 vs. 2673 CPM), but not for vigorous intensity (7148 vs. 3469 CPM). All the developed cut‐points is shown in with a red (moderate) and blue (vigorous) dotted line in Figures 2 and 3.

**TABLE 2:** ENMO and AGVMC cut‐points for moderate and vigorous intensity based on ROC analysis for hip (left), hip (right), dominant and non‐dominant wrist, thigh, and lower back worn accelerometers (*n* = 50).

	Moderate				Vigorous			
	Se	Sp	AUC	Cut‐points	Se	Sp	AUC	Cut‐points
**ENMO (mg)**								
Hip (left)	85	82	0.90	23	87	86	0.93	107
Hip (right)	79	86	0.89	29	89	83	0.93	99
Lower back	80	86	0.90	26	85	89	0.93	112
Thigh	80	83	0.87	47	80	89	0.91	207
Dominant wrist	67	76	0.75	84	81	79	0.81	90
Non‐dominant wrist	75	78	0.81	76	90	76	0.87	84
**AGVMC (CPM)**								
Hip (left)	83	78	0.87	2343	74	85	0.87	3046
Hip (right)	84	78	0.87	2299	91	70	0.84	2681
Lower back	78	83	0.87	2673	74	85	0.87	3569
Thigh	82	83	0.88	2794	80	85	0.89	7148
Dominant wrist	30	97	0.59	9267	46	95	0.69	9655
Non‐dominant wrist	53	64	0.54	5978	38	84	0.57	7415

Abbreviations: AGVMC = actigraph vector magnitude counts per minute, AUC = area under the curve, CPM = Counts per minute, ENMO = Euclidean norm minus one, mg = milli gravity, ROC = receive operating characteristics, Se = sensitivity, Sp = specificity.

### 4.4 Moderate and Vigorous Intensity Classification Using ML Models

The accuracy of the ML models to predict PA intensity in the training sample is presented in Table 3. The performance was generally high across all anatomical placements, with left hip showing the highest accuracy (0.89) and non‐dominant wrist the lowest accuracy (0.83). A similar pattern was observed for sensitivity and specificity across all six anatomical placements, with the left hip showing the highest sensitivity (0.87) and specificity (0.93) along with the non‐dominant wrist showing the lowest sensitivity (0.83) and specificity (0.90).

**TABLE 3:** Performance of ML models for PA intensity classification for hip (left), hip (right), dominant and non‐dominant wrist, thigh, and lower back worn accelerometers (*n* = 50).

	Sensitivity	Specificity	Accuracy
Hip (left)	0.87	0.93	0.89
Hip (right)	0.86	0.92	0.87
Lower back	0.83	0.90	0.85
Thigh	0.84	0.91	0.86
Dominant wrist	0.84	0.90	0.84
Non‐dominant wrist	0.83	0.90	0.83

The feature importance for the random forest models across different anatomical placements is detailed in the Figure S3. For left and right hip, lower back, and thigh, the standard deviation of the acceleration in single axis or vector emerged as the key features for classifying PA intensity. In contrast, the dominant and non‐dominant wrist exhibited different pattern, with the mean accelerometer values of single axis, ENMO, and the standard deviation of inclination being the most significant features.

### 4.5 Validation of Moderate and Vigorous Intensity Cut‐Points and ML Models

Validation of the developed cut‐points and ML models to predict PA intensity in the hold‐out validation sample is shown in Tables 4 and 5. In Table 4 moderate and vigorous ENMO cut‐points from ROC analysis were used to predict PA intensity and validate against %V̇O_2_R classification. Similar, the random forest models were validated against %V̇O_2_R classification (Table 5). The ENMO cut‐points derived from the ROC analysis showed similar overall accuracy (i.e., 0.62–0.63) across hip (right and left), thigh, and lower back (Table 4). From wrist worn accelerometer, the overall accuracy was lower for both dominant (0.57) and non‐dominant (0.57). The class specific sensitivity, specificity, precision, and F1‐score were similar across hip (right and left), thigh and lower back. The sensitivity was better for light and vigorous intensity compared to moderate intensity for all six accelerometers. The random forest model based on accelerometer features combined with simple participants characteristics improved the overall accuracy of the prediction of PA intensity in all six positions (Table 5) ranging from 0.66 for the right hip and lower back to 0.71 for the thigh accelerometer. The prediction of moderate intensity was better compared to the ROC developed cut‐points and highest in the thigh. However, prediction of the moderate category was still lower compared to light and vigorous. A secondary analysis was performed to address the challenge in differentiating between moderate and vigorous intensities. We thereby combined moderate and vigorous into a single category, MVPA. The results showed an increase in accuracy across all anatomical locations from 0.76 to 0.80 (Table S5).

**TABLE 4:** Validation of ROC analysis cut‐points for PA intensity classification for hip (left), hip (right), dominant and non‐dominant wrist, thigh, and lower back worn accelerometers using the ENMO metric in a hold‐out sample (*n* = 26).

	Sensitivity (recall)	Specificity	Precision	F1‐score	Accuracy
**Hip (left)**					0.63
Light	0.74	0.77	0.67	0.70	
Moderate	0.32	0.82	0.45	0.37	
Vigorous	0.80	0.84	0.69	0.74	
**Hip (right)**					0.62
Light	0.82	0.72	0.65	0.73	
Moderate	0.19	0.8	0.43	0.26	
Vigorous	0.80	0.81	0.65	0.72	
**Lower back**					0.60
Light	0.78	0.73	0.66	0.71	
Moderate	0.20	0.81	0.32	0.24	
Vigorous	0.77	0.84	0.68	0.72	
**Thigh**					0.61
Light	0.78	0.75	0.68	0.73	
Moderate	0.31	0.79	0.38	0.34	
Vigorous	0.67	0.87	0.69	0.68	
**Dominant wrist**					0.57
Light	0.77	0.58	0.56	0.65	
Moderate	0.10	0.93	0.38	0.16	
Vigorous	0.80	0.82	0.56	0.66	
**Non‐dominant wrist**					0.57
Light	0.76	0.67	0.60	0.67	
Moderate	0.10	0.91	0.33	0.1	
Vigorous	0.79	0.75	0.51	0.68	

Abbreviations: ENMO = Euclidean norm minus one; ROC = receive operating characteristics.

**TABLE 5:** Validation of ML models for PA intensity classification for hip (left), hip (right), dominant and non‐dominant wrist, thigh, and lower back worn accelerometers in a hold‐out sample (*n* = 26).

	Sensitivity (recall)	Specificity	Precision	F1‐score	Accuracy
**Hip (left)**					0.67
Light	0.87	0.70	0.71	0.78	
Moderate	0.34	0.83	0.47	0.39	
Vigorous	0.70	0.93	0.77	0.73	
**Hip (right)**					0.66
Light	0.88	0.68	0.70	0.78	
Moderate	0.29	0.82	0.41	0.34	
Vigorous	0.70	0.94	0.79	0.74	
**Lower back**					0.66
Light	0.88	0.71	0.72	0.79	
Moderate	0.31	0.84	0.45	0.37	
Vigorous	0.69	0.91	0.72	0.71	
**Thigh**					0.71
Light	0.89	0.74	0.75	0.82	
Moderate	0.39	0.85	0.49	0.43	
Vigorous	0.71	0.94	0.79	0.75	
**Dominant wrist**					0.69
Light	0.88	0.68	0.70	0.78	
Moderate	0.37	0.84	0.49	0.43	
Vigorous	0.72	0.97	0.89	0.79	
**Non‐dominant wrist**					0.68
Light	0.87	0.70	0.71	0.78	
Moderate	0.33	0.86	0.51	0.40	
Vigorous	0.73	0.92	0.74	0.73	

## 5 Discussion

This is the first study to provide accelerometer cut‐points and ML models for classifying moderate and vigorous PA intensities for six anatomical locations in older adults (75+ years). The study (1) synchronizes accelerometers with direct oxygen uptake during daily activities of different metabolic intensities, (2) applies V̇O_2_R to account for age‐related changes in RMR and VO_2_max, (3) uses ROC and ML methodologies to integrate accelerometer features and participant characteristics, and (4) validates the ROC developed cut‐points and ML models against VO_2_R using a hold‐out sample.

As age increases, both RMR and VO_2_max decline [4, 11], leading to a reduced cardiovascular reserve (VO_2_R) as demonstrated for walking [13]. These factors are important to consider, as they may have substantial impact in classifying PA intensity [19]. Validation studies with the goal to precisely classifying PA intensities, particularly in the oldest old, thereby should address changes in RMR and VO_2_max. A recent narrative review has shown that even small doses of vigorous activity reduce the risk of morbidity and mortality [3], highlighting the importance of classifying both moderate and vigorous PA intensity. Furthermore, this classification is crucial for accurately evaluating the adherence to PA guidelines in the aging population [2].

The main findings of the study were that for both the ENMO and AGVMC metrics, the cut‐points developed with ROC analysis for the left and right hip, lower back, and thigh demonstrated good to excellent AUC values, ranging from 0.84 to 0.93 (Table 2). Based on our knowledge, these are the first cut‐points established for the thigh and lower back positions in older adults. The cut‐points for dominant and non‐dominant wrists showed lower AUC values compared to the other positions. The accuracy of the hip‐ and wrist cut‐points in the current study are comparable to other validation studies in older adults [8, 9, 17].

The ML models demonstrated strong performance across all anatomical placements, with accuracy ranging from 0.83 to 0.89. These models significantly improved the classification of PA intensity for both dominant and non‐dominant wrist when compared to ROC analysis. The accuracy is similar to that of random forest models in another study on older adults, which used binary intensity classification (e.g., moderate vs. non‐moderate), METs as criterion metric and the right wrist [34]. In our study, we use VO_2_R as the criterion and provide models for six different accelerometer wear positions. Interestingly, the most important features in the ML models varied depending on the wear location of the accelerometers. For the right and left hip, lower back, and thigh placements, the most important variables included the standard deviation of acceleration in a single axis or vector value as well as maximal standard deviation of the acceleration (Figure S3). The importance of the standard deviation of the acceleration at these anatomical sites likely reflects its relationship to ground reaction force, which previous has been linked to the amplitude of raw acceleration signals from the hip position during different walking and running speeds [35, 36]. Furthermore, energy expenditure during locomotion has shown to increase with increasing speed, along with greater acceleration output from accelerometers, highlighting the association between mechanical output and metabolic cost [37]. As the acceleration from left and right hip, thigh and lower back were relatively low during the specific daily activities in ADL 1 and ADL 2, the main acceleration in these positions may be represented by the acceleration from walking between daily activities in the ADL circuits and from the two 6‐MWTs (Figure 2). However, for the dominant and non‐dominant wrist positions mean acceleration, ENMO and standard deviation of the inclination angle were important features. This likely reflects the greater influence of upper body movement on wrist accelerations during the two ADL circuits (Figure 2), while the lower body remained relatively stationary, resulting in a less pronounced ground reaction force and lower energy cost. These findings highlight the distinct influence of wear location on the relevant features for PA intensity classification, particularly when considering daily and walking activities.

### 5.1 Comparison to the Literature

#### 5.1.1 Hip Accelerometer (Right Position)

In our study, the hip‐worn (right) accelerometer cut‐points for the ENMO variable were 29 mg for moderate and 99 mg for vigorous intensity. Our moderate cut‐point was lower than two previous studies (82 and 55 mg) [16, 17], but higher than one study (14 mg) [8]. These differences may be due activities included in the protocol [38] and the choice of criterion measure [19]. For example, Migueles et al. [8] used METs and included sedentary activities, while our study used VO_2_R and focused on light to vigorous activities. To the best of our knowledge, only one study has established ENMO cut‐points for vigorous intensity [16] in relatively younger older adults (62.9 ± 3.6 years). The cut‐point identified was substantially lower than the cut‐point determined in our study (99 vs. 191 mg) [16]. This may be explained by the difference in criterion measure used. Bammann et al. (2021) used the METs metric as criterion, which does not account for the age‐related changes in VO_2_max. The VO_2_R methodology used in our study incorporates both age‐related changes in RMR and VO_2_max [19].

The AGVMC cut‐points for moderate and vigorous in the right hip position were 2299 and 2681 CPM, respectively. Our moderate cut‐point was higher than one study (1924 CPM) [5], but lower compared to two other studies (2858 CPM and 2751 CPM) [16, 18]. Similar to ENMO, the vigorous cut‐point for AGVMC in our study was significant lower compared to two other studies (2681 vs. 6799 CPM and 9359 CPM) [16, 18], which both applied the METs metric to establish the cut‐points. Furthermore, these studies established cut‐points in a younger group of older adults, with average ages of 62.9 ± 3.6 and 71.9 ± 5.4 years.

This suggests that applying the vigorous cut‐points for both ENMO and AGVMC from our study would result in an increased estimate of time spent in vigorous intensity.

#### 5.1.2 Wrist Accelerometer

The dominant and non‐dominant wrist cut‐points applying both ENMO and AGVMC failed to effectively differentiate between moderate and vigorous intensity levels as the ROC analysis provided similar cut‐points for both intensities (Table 2). This indicates that the acceleration from the dominant and non‐dominant wrist accelerometers did not increase proportionally with the increase in the %VO_2_R across the four activities included in the study (Figures 1, 2, 3). This may suggest that wrist accelerometers have difficulties differentiating between moderate and vigorous activity levels when PA consists of a mix of daily and walking activities and when ROC analysis is used. This is a highly relevant point to consider since for many old and very old adults carrying out daily activities at home represent a considerable amount of the daily PA.

Overall, the ROC established intensity cut‐points showed similar accuracy as other validation studies in older adults. However, the novelty in the current study was to include a very old population, multiple anatomical positions, and using VO_2_R as a criterion. These cut‐points consider the age‐related changes in RMR and VO_2_max and allows for comparability across studies using different anatomical positions in the very old adults.

### 5.2 Hold‐Out Sample Validation and Comparison Between ROC Analysis and ML Models

A hold‐out validation can provide insights into the precision and applicability of cut‐points and ML models when applied to other cohort studies [39]. Few earlier studies have applied this approach in older adults using a binary variable which categorizes activity as below or above the developed threshold (e.g., moderate intensity) [8, 17]. We used a multiclass method (i.e., light, moderate, and vigorous), which may add important information in evaluating free‐living PA data. Our results indicate that when validating the ENMO cut‐points against VO_2_R in a hold‐out sample, accuracy ranged from 0.56 to 0.63, with the dominant and non‐dominant wrist showing the lowest accuracy and the left hip the highest (Table 4). This is notably higher compared to a recent study with a similar approach, which reported low intra‐class correlation (ICC) values (0.17–0.40) for the intensity classification (i.e., light/moderate/vigorous) between the criterion (VO_2_) and hip and wrist accelerometer cut‐points in older adults during activities such as reading, ADL, cycling, and aerobics [16]. Importantly the ML models in the current study, improved the accuracy of PA intensity classification, with the thigh‐worn accelerometer showing the highest accuracy (0.71), and the right hip and lower back the lowest (0.66) (Table 5). Furthermore, the ML models markedly increased the accuracy of PA intensity prediction for dominant and non‐dominant wrist cut‐points, when compared to ROC analysis. This is particularly important as larger cohort studies, such as UK biobank, incorporate only wrist accelerometers [40]. Another interesting finding was that light and vigorous intensity showed higher sensitivity across all anatomical placements compared to moderate (Tables 4 and 5). A possible explanation for the overall lower sensitivity of moderate intensity may be the mismatch between the proportional increase across the four included activities in the %VO_2_R and acceleration (Figures 1, 2, 3). Particularly, the relative increase in the acceleration between ADL 1 and the 6‐MWTs was significantly higher than the relative increase in %VO_2_R. Both ADL and walking are part of older individuals' daily lives, and it is therefore crucial to incorporate these activities into validation studies for older adults. However, no studies have yet developed an ideal model to distinguish between these daily activities and accurate predict PA intensity in this population. Another possible explanation for the lower moderate sensitivity may be due to the reduced cardiovascular reserve (VO_2_R), which indicate a lower range between moderate and vigorous intensity. Furthermore, it has previous been demonstrated that metabolic efficiency is reduced with increasing age [41]. This may further affect the classification of PA intensity and represent an additional methodological challenge when using accelerometry methodology as changes in movement (acceleration) might decrease, but energy cost may increase with age, as shown for walking [13]. This may imply that categorizing moderate and vigorous intensity in very old adults with low VO_2_max, and consequently lower VO_2_R, can be challenging.

The challenge in differentiating between moderate and vigorous intensities was further investigated in secondary analysis using random forest models merging moderate and vigorous intensity into MVPA. The results demonstrated an increase in accuracy (0.76–0.80) across all anatomical locations (Table S5). Our data demonstrated higher sensitivity compared to a recent study using ENMO, with values of 0.81 versus 0.70 for the hip and 0.87 versus 0.83 for the non‐dominant wrist [8]. However, specificity was lower in our project, measuring 0.72 versus 0.99 for the hip and 0.75 versus 0.99 for the non‐dominant wrist [8]. This may be due to the relatively low cut‐points established by Migueles and colleagues, which may not only increase the likelihood of true negatives but also increase the risk of misclassifying moderate‐intensity. Our models build on the cut‐points developed by Migueles, using VO_2_R as the criterion and considering age, sex, and BMI in the PA intensity classification.

### 5.3 Strength and Limitations

The main strength of this study lies in the novel establishment of moderate and vigorous PA cut‐points using the VO_2_R metric as criterion. This contributes to existing knowledge, which is predominantly based on estimated METs or, in some cases, measured METs without accounting for age‐related changes in VO_2_max. This may offer a more accurate comparison of PA intensity across age‐groups compared to the METs metric [19].

The use of accelerometers on six different anatomical placements synchronized during the same protocol is another important strength and it allows for better comparison among studies, which used different anatomical placements. Additionally, our study included a relatively old population (75 years and above) as opposed to most existing validation studies conducted in younger older adults [5, 9, 16, 17, 18]. Furthermore, our protocol included common ADL in circuits which are routinely carried out at home and often represent a great proportion of everyday PA in old and very old adults. The collection of multiple ADLs in circuits is also a strength as it may mimic everyday life to larger degree compared to measurement of single daily activities.

This study has some limitations. Despite the fact that we enrolled relatively old adults (80.2 ± 3.7 years), the physical function tests (SPPB and 6‐MWT) indicated that our participants were relatively well‐functioning [31]. This may limit generalizability to frailer groups, but it also highlights that the misclassification of PA intensity may be even larger in these groups. Another potential limitation lies in the methodological approach to average oxygen uptake during ADL Circuit 1 and ADL Circuit 2, which combines standing and short walking activities. However, our criteria were to mimic a real‐life scenario and to ensure a steady state during each circuit and not during each specific activity. Third, incorporating sedentary activities in the protocol to establish sedentary behavior cut‐points would have enhanced the intensity classification models, by enabling the possibility to classify sedentary behavior and light PA. Finally, using different brands across wear sites may have influenced the classification. However, a recent study has shown good to excellent intra class correlations between intensity gradients between the three included accelerometer brands [26].

## 6 Conclusion

In conclusion, the study provides accelerometer cut‐points and ML models for classifying moderate and vigorous PA intensities for hip (left and right), wrist (dominant and non‐dominant), thigh, and lower back in very old adults. By using VO_2_R as criterion, the study accounts for the age‐related changes in RMR and VO_2_max. The developed ML models demonstrated higher accuracy compared to ROC analysis developed cut‐points and showed similar accuracy across the six different anatomical placements which allows for comparability across studies with different anatomical placement of the accelerometer. The ML model from the thigh showed the highest overall accuracy for classifying PA intensity during a mix of daily and walking activities indicating this as a potentially preferable anatomic location for collecting accelerometry PA for old and very old adults.

## 7 Perspectives

This study contributes to the existing knowledge and understanding of accelerometer‐measured PA intensity classification in older adults by demonstrating better accuracy of PA intensity classification in a hold‐out sample compared to a recent study showing low ICC for PA intensity using a multiclass approach [16]. The higher accuracy of ML models and lower vigorous cut‐points may significantly impact future research on accelerometer‐measured PA in older adults. Using our models will likely result in higher estimates of vigorous intensity, suggesting that older adults may not be as inactive as currently believed. The results from the study also highlight the complexity of accurately classifying moderate and vigorous intensity with accelerometry in older adults based on current methodologies as the sensitivity of moderate intensity was relatively low when validation the commonly developed ROC analysis cut‐points and ML models in the current study. Further studies should aim to develop models that enhance the sensitivity of moderate intensity. Additionally, they should consider age‐related changes in RMR and VO_2_max and provide external validation using a multiclass approach.

## Conflicts of Interest

The authors declare no conflicts of interest.

## Supporting information


Data S1.

## Data Availability

The random forest models developed in this study for classifying PA intensity—both moderate and vigorous and overall MVPA—in very old adults are available in the online repository for each anatomical placement: https://github.com/mskjodt/PA‐classification‐models. These models provide a valuable tool for researchers aiming to assess moderate and vigorous intensity in the older population.
