# Supplementary material for: Moderate and Vigorous Physical Activity Intensity Cut‐Points for Hip‐, Wrist‐, Thigh‐, and Lower Back Worn Accelerometer in Very Old Adults
Source: Scand J Med Sci Sports. 2025 Jan 3;35(1):e70009. doi: 10.1111/sms.70009 (PMC11698702; doi:10.1111/sms.70009)
Supplement: Supplementary file 1 — Data S1. [file SMS-35-e70009-s001.docx]

**Supplemental material**

**eTable 1 – Features used in the random forest model**

**eTable 2 - Average values of VO_2_, %VO_2_R, mean acceleration from MAD and AI across the four activities and accelerometer placement (n=77)**

**eTable 3 - MAD and AI cut-points for moderate and vigorous intensity based on ROC analysis for hip (left), hip (right), dominant and non-dominant wrist, thigh and lower back worn accelerometers using VO_2_R as criterion (n=51)**

**eTable 4 – Cross-validation of ROC analysis cut-points for physical activity intensity classification for hip (left), hip (right), dominant and non-dominant wrist, thigh, and lower back worn accelerometers using the AGVMC metric (n=26)**

**eFigure 1 – Correlations between ENMO metric for different anatomical placements (n=77)**

**eFigure 2 – Correlations between AGVMC metric for different anatomical placements (n=77)**

**eTable 5 – Cross-validation of random forest models for light and MVPA intensity classification for hip (left), hip (right), dominant and non-dominant wrist, thigh, and lower back worn accelerometers (n=26)**

**eFigure 3 – Feature importance for the random forest models across all six anatomical placements**

**eTable 1 – Features used in the random forest model**

| **ENMO** | Euclidean Norm Minus One |
| --- | --- |
| **maccX** | Mean acceleration in the x axis |
| **maccY** | Mean acceleration in the y axis |
| **maccZ** | Mean acceleration in the z axis |
| **sdaccX** | Standard deviation of the acceleration in the x axis |
| **sdaccY** | Standard deviation of the acceleration in the y axis |
| **sdaccZ** | Standard deviation of the acceleration in the z axis |
| **mean** | Mean acceleration of the vector values |
| **sd** | Standard deviation of the acceleration of the vector values |
| **sdmax** | Maximal standard deviation |
| **incl** | Mean inclination angle |
| **angl** | Mean angle |
| **sdincl** | Standard deviation of the inclination angle |
| **sdangl** | Standard deviation of the angle |
| **age** | Age of the participants |
| **sex** | Sex of the participants |
| **bmi** | Body Mass Index of the participants |

**eTable 2 - Average values of VO_2_, %VO_2_R, mean acceleration from MAD and AI across the four activities and accelerometer placement (n=77)**

|  | **VO_2_** | **%VO_2_R** | **Hip (left)** | **Hip (right)** | **Lower back** | **Thigh** | **Dominant wrist** | **Non-dominant wrist** |
| --- | --- | --- | --- | --- | --- | --- | --- | --- |
| **MAD** | O_2_·min^-1^·kg^-1^ | % | mg | mg | mg | mg | mg | mg |
|  | Mean (SD) | Mean (SD) | Mean (SD) | Mean (SD) | Mean (SD) | Mean (SD) | Mean (SD) | Mean (SD) |
| **ADL Circuit 1** | 8.9 ± 1.3 | 30 ± 8 | 32.1 ± 12.7 | 31.7 ± 12.5 | 31.1 ± 12.0 | 54.4 ± 26.6 | 132 ± 41 | 116 ± 39 |
| **ADL Circuit 2** | 10.7 ± 1.6 | 39 ± 10 | 41.2 ± 11.9 | 41.1 ± 11.8 | 40.1 ± 11.9 | 67.5 ± 23.8 | 153 ± 41 | 136 ± 44 |
| **6-MWT (Self-selected)** | 12.9 ± 1.8 | 50 ± 11 | 200 ± 51 | 198 ± 51 | 200 ± 51 | 378 ± 99 | 163 ± 37 | 179 ± 43 |
| **6-MWT (Maximal)** | 18.6 ± 3.4 | 76 ± 13 | 323 ± 84 | 318 ± 82 | 331 ± 82 | 557 ± 147 | 236 ± 68 | 242 ± 65 |
|  | **VO_2_** | **%VO_2_R** | **Hip (left)** | **Hip (right)** | **Lower back** | **Thigh** | **Dominant wrist** | **Non-dominant wrist** |
| **AI** | O_2_·min^-1^·kg^-1^ | % | mg | mg | mg | mg | mg | mg |
|  | Mean (SD) | Mean (SD) | Mean (SD) | Mean (SD) | Mean (SD) | Mean (SD) | Mean (SD) | Mean (SD) |
| **ADL Circuit 1** | 8.9 ± 1.3 | 30 ± 8 | 342 ± 95 | 343 ± 90 | 339 ± 89 | 569 ± 202 | 1225 ± 318 | 1091 ± 325 |
| **ADL Circuit 2** | 10.7 ± 1.6 | 39 ± 10 | 440 ± 99 | 455 ± 99 | 457 ± 102 | 718 ± 190 | 1269 ± 306 | 1165 ± 340 |
| **6-MWT (Self-selected)** | 12.9 ± 1.8 | 50 ± 11 | 1130 ± 242 | 1112 ± 233 | 1095 ± 230 | 2800 ± 690 | 1016 ± 217 | 1009 ± 205 |
| **6-MWT (Maximal)** | 18.6 ± 3.4 | 76 ± 13 | 1730 ± 413 | 1690 ± 401 | 1735 ± 390 | 4156 ± 1106 | 1734 ± 553 | 1543 ± 451 |

SD=Standard Deviation. ADL=Activities of Daily Living, 6-MWT – Self=6-Minutes-Walking-Test at self-selected gait speed. 6-MWT – Max=6-Minutes-Walking-Test at maximal gait speed, mg=milli gravity, MAD= Mean Amplitude Deviation, AI= Activity Index

**eTable 3 - MAD and AI cut-points for moderate and vigorous intensity based on ROC analysis for hip (left), hip (right), dominant and non-dominant wrist, thigh and lower back worn accelerometers using VO_2_R as criterion (n=51)**

|  | **Moderate** | | | | **Vigorous** | | | |
| --- | --- | --- | --- | --- | --- | --- | --- | --- |
|  | **Se** | **Sp** | **AUC** | **Cut-points** | **Se** | **Sp** | **AUC** | **Cut-points** |
| **MAD (mg)** |  |  |  |  |  |  |  |  |
| Hip (left) | 85 | 82 | 0.90 | 45 | 89 | 84 | 0.93 | 212 |
| Hip (right) | 79 | 86 | 0.89 | 57 | 81 | 91 | 0.93 | 229 |
| Lower back | 80 | 86 | 0.90 | 52 | 85 | 88 | 0.93 | 221 |
| Thigh | 78 | 86 | 0.88 | 110 | 80 | 90 | 0.92 | 414 |
| Dominant Wrist | 59 | 84 | 0.75 | 174 | 78 | 81 | 0.81 | 180 |
| Non-dominant wrist | 76 | 78 | 0.81 | 150 | 90 | 76 | 0.87 | 167 |
| **AI (CPM)** |  |  |  |  |  |  |  |  |
| Hip (left) | 81 | 85 | 0.90 | 570 | 85 | 87 | 0.93 | 1218 |
| Hip (right) | 81 | 85 | 0.90 | 543 | 89 | 84 | 0.93 | 1128 |
| Lower back | 86 | 83 | 0.90 | 505 | 87 | 88 | 0.94 | 1156 |
| Thigh | 79 | 86 | 0.88 | 1031 | 91 | 79 | 0.91 | 2280 |
| Dominant Wrist | 31 | 95 | 0.61 | 1519 | 54 | 92 | 0.72 | 1519 |
| Non-dominant wrist | 37 | 96 | 0.63 | 1363 | 58 | 91 | 0.78 | 1369 |

ROC=Receive Operating Characteristics, Se=Sensitivity, Sp=Specificity, AUC=Area under the curve, MAD= Mean Amplitude Deviation, AI= Activity Index

**Table 4 –Validation of ROC analysis cut-points for physical activity intensity classification for hip (left), hip (right), dominant and non-dominant wrist, thigh, and lower back worn accelerometers using the AGVMC metric (n=26)**

|  | **Sensitivity (recall)** | **Specificity** | **Precision** | **F1-score** | **Accuracy** |
| --- | --- | --- | --- | --- | --- |
| **Hip (left)** |  |  |  |  | 0.57 |
| Light | 0.75 | 0.63 | 0.63 | 0.69 |  |
| Moderate | 0.11 | 0.91 | 0.34 | 0.17 |  |
| Vigorous | 0.79 | 0.78 | 0.54 | 0.64 |  |
| **Hip (right)** |  |  |  |  | 0.57 |
| Light | 0.78 | 0.66 | 0.66 | 0.71 |  |
| Moderate | 0.06 | 0.95 | 0.33 | 0.10 |  |
| Vigorous | 0.81 | 0.72 | 0.49 | 0.61 |  |
| **Lower back** |  |  |  |  | 0.58 |
| Light | 0.78 | 0.63 | 0.65 | 0.71 |  |
| Moderate | 0.21 | 0.85 | 0.36 | 0.26 |  |
| Vigorous | 0.63 | 0.85 | 0.56 | 0.60 |  |
| **Thigh** |  |  |  |  | 0.65 |
| Light | 0.79 | 0.77 | 0.76 | 0.77 |  |
| Moderate | 0.35 | 0.81 | 0.40 | 0.37 |  |
| Vigorous | 0.71 | 0.88 | 0.66 | 0.68 |  |
| **Dominant Wrist** |  |  |  |  | 0.52 |
| Light | 0.89 | 0.23 | 0.51 | 0.65 |  |
| Moderate | 0.04 | 0.97 | 0.36 | 0.07 |  |
| Vigorous | 0.38 | 0.93 | 0.64 | 0.48 |  |
| **Non-dominant wrist** |  |  |  |  | 0.34 |
| Light | 0.45 | 0.45 | 0.42 | 0.43 |  |
| Moderate | 0.16 | 0.74 | 0.20 | 0.18 |  |
| Vigorous | 0.36 | 0.77 | 0.34 | 0.35 |  |

ROC=Receive Operating Characteristics**,** AGVMC=Actigraph vector magnitude counts

**eFigure 1 – Correlations between ENMO metric for different anatomical placements (n=77)**

**
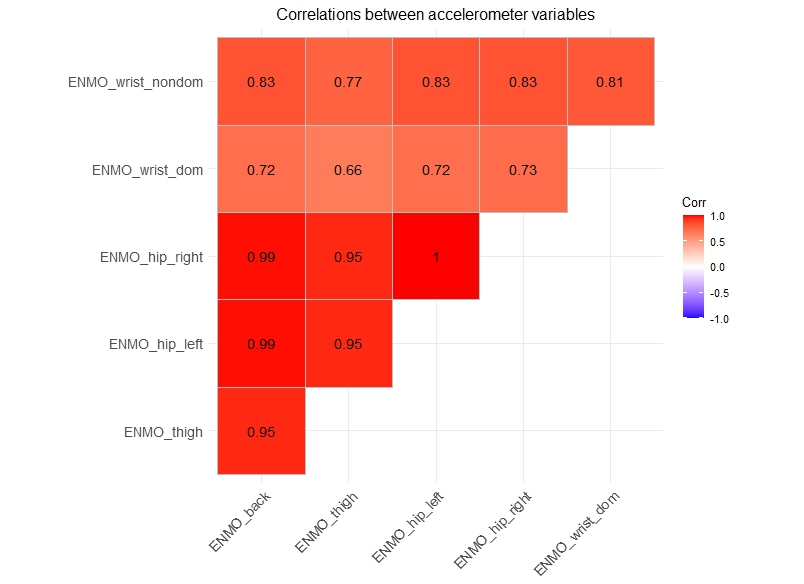
**

ENMO=Euclidean Norm Minus One

**eFigure 2 – Correlations between AGVMC metric for different anatomical placements (n=77)**

**
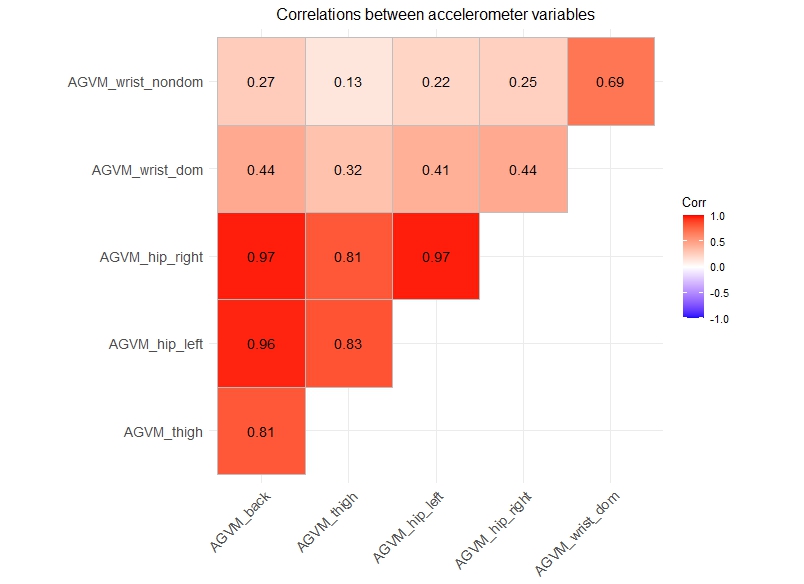
**

AGVMC=Actigraph vector magnitude counts

**eTable 5 – Validation of random forest models for light and MVPA intensity classification for hip (left), hip (right), dominant and non-dominant wrist, thigh, and lower back worn accelerometers (n=26)**

|  | **Sensitivity (recall)** | **Specificity** | **Precision** | **F1-score** | **Accuracy** |
| --- | --- | --- | --- | --- | --- |
| **Hip (left)** | 87 | 70 | 71 | 78 | 0.78 |
| **Hip (right)** | 81 | 72 | 70 | 75 | 0.76 |
| **Lower back** | 85 | 77 | 76 | 80 | 0.81 |
| **Thigh** | 87 | 74 | 75 | 81 | 0.80 |
| **Dominant Wrist** | 86 | 71 | 72 | 78 | 0.78 |
| **Non-dominant wrist** | 87 | 75 | 74 | 80 | 0.81 |

**eFigure 3 – Feature importance for the random forest models across all six anatomical placements**

**
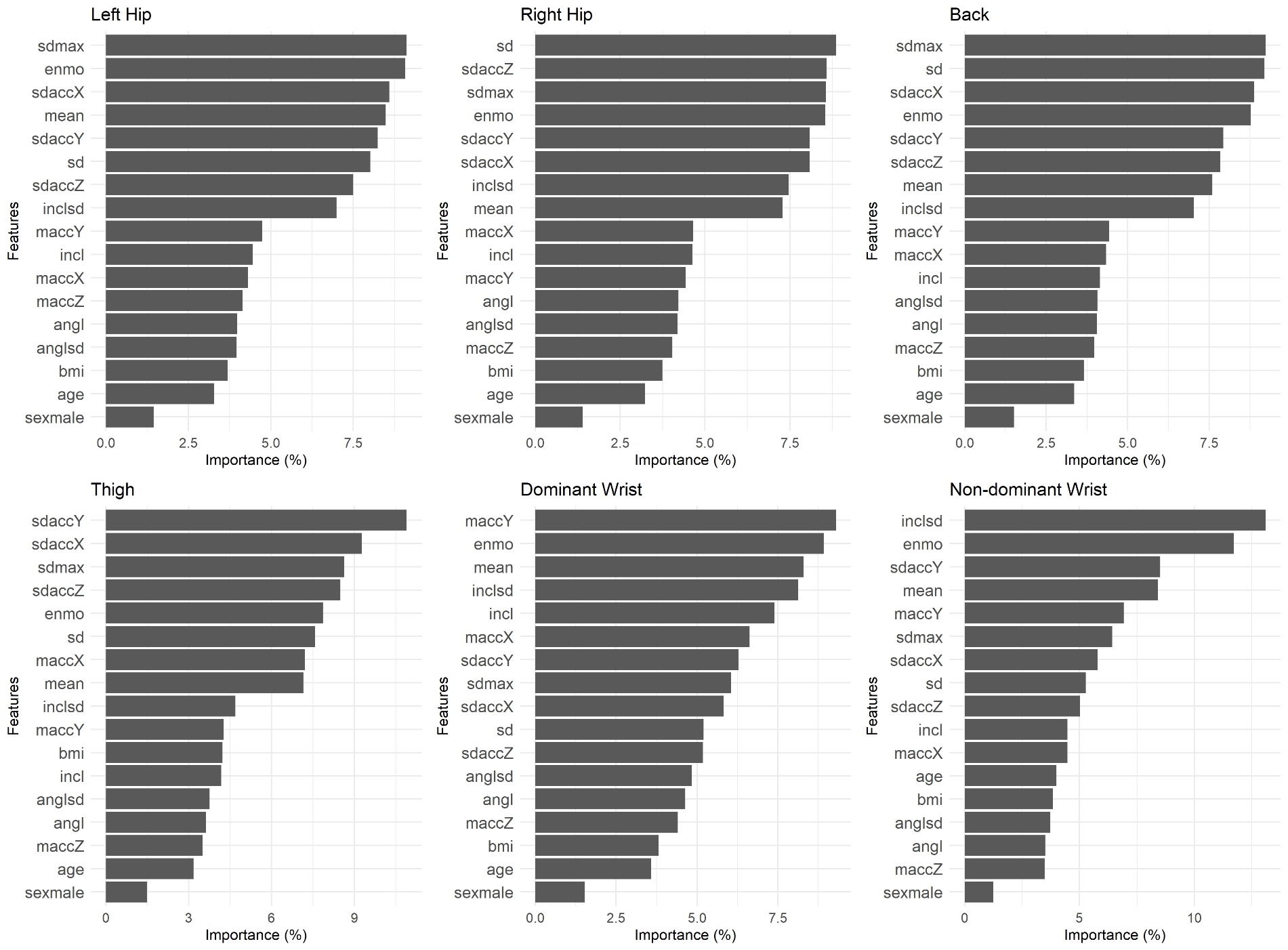
**
